# Anaesthesiologists’ perspectives of the need for nurse anaesthetists in South Africa

**DOI:** 10.4102/curationis.v48i1.2587

**Published:** 2025-04-30

**Authors:** Kalonji K. Yezu, Charlene Downing, Sidwell Matlala

**Affiliations:** 1 Department of Nursing, Faculty of Health Sciences, University of Johannesburg, Johannesburg, South Africa

**Keywords:** Anaesthesiologist shortage, nurse anaesthetists, South African healthcare, anaesthesia services, healthcare workforce solutions

## Abstract

**Background:**

South Africa faces a significant shortage of anaesthesiologists, compromising healthcare access and increasing anaesthesia-related risks. Addressing this issue requires sustainable, locally relevant solutions aligned with global surgery initiatives to enhance surgical care access.

**Objectives:**

This study explores anaesthesiologists’ perspectives on the necessity of nurse anaesthetists in South Africa and offers recommendations for developing their practice.

**Method:**

A qualitative, descriptive, and contextual design was employed, using purposive and snowball sampling methods. The population consisted of anaesthesiologists registered with the Health Professions Council of South Africa (HPCSA). Data were collected through in-depth, semi-structured interviews conducted face-to-face and online. Analysis followed Colaizzi’s seven-step method, supported by an independent coder.

**Results:**

Findings revealed diverse perspectives, ranging from support for independent, well-trained nurse anaesthetists under supervision or as assistants, to complete opposition to nurse anaesthetists administering anaesthesia. Participants emphasised that the anaesthesiology specialist community should define nurse anaesthetists’ scope of practice, which must be regulated. Training should align with diplomate anaesthetists’ standards and involve anaesthesiologists supported by nursing educators.

**Conclusion:**

This pioneering research addresses a critical gap in South Africa’s healthcare system by exploring the introduction of nurse anaesthetists to mitigate the shortage of anaesthesia providers.

**Contribution:**

Its findings hold the potential to inform policy and practice, contributing to the advancement of anaesthesia services and addressing a pressing healthcare need in South Africa.

## Introduction

Essential and emergency surgical care is vital to universal health coverage. In 2015, the 68th World Health Assembly of the World Health Organization (WHO) acknowledged anaesthesia services as a vital component in strengthening essential and emergency surgical care. It is reported that one-third of the global disease burden requires surgical and anaesthesia services (Reddy, Makasa & Biccard 2019:624). However, inadequate access to these services – because of a global shortage of anaesthesia providers – currently affects five billion people worldwide and accounts for 143 million surgical procedure postponements each year, 17 million preventable deaths and life-long disabilities (Rickard et al.2020:483). This has led to urgent calls to develop viable anaesthesia care models worldwide to increase access to anaesthesia services for most of the global population (Wollner et al. 2020:924).

The shortage of anaesthesia providers is more severe in developing countries, in particular Africa and Southeast Asia, which account for 48% of the world’s population, but only 15% of the global anaesthesiology workforce (Turkot & Banks 2019:1). Anaesthesia is especially critical in obstetrical instances, and an estimated 808 women die every day as a result of pregnancy and delivery difficulties that necessitate surgical intervention (WHO 2017:1). Many countries in Africa, including South Africa, are below the World Federation of Societies of Anaesthesiologists’ (WFSA) recommended target of five anaesthesiologists per 100 000 people (Tao, Sokha & Yuan 2020:2). This means that African countries have an urgent need for anaesthesia providers to alleviate surgical care backlogs, preventable deaths and preventable life-long disabilities (Vreede, Bulamba & Chikumba 2019:1199).

Countries such as Ghana, Sierra Leone, Uganda and Somaliland have established initiatives to increase access to safe anaesthesia care by task-shifting and training non-physician anaesthetists (NPAs), sometimes known as non-physician anaesthesia providers (NPAPs), to administer certain forms of anaesthesia (Edgcombe et al. 2019:2). These NPAs are qualified health professionals like licensed nurse anaesthetists with a full scope of practice as anaesthesia providers in some settings (Edgcombe et al. 2019:1). Non-physician anaesthetists can deliver safe anaesthesia care, potentially helping to prevent the 17 million deaths each year attributed to surgically treatable conditions (Bath, Bashford & Fitzgerald 2019:3). These NPAs thus constitute a large proportion of the global anaesthesia workforce, particularly in resource-limited countries (WFSA 2021:2).

Nurse anaesthetists fall under the category of NPAs and are recognised as one possible solution to the anaesthesia workforce shortage in Africa (Barash & Newton 2018:1). These individuals are qualified clinical nurse specialists who deliver anaesthesia, respiratory treatment, cardiopulmonary resuscitation or other emergency life-sustaining services (American Association of Nurse Anaesthetists 2021:1). There has been an increased need for nurses to provide anaesthetic treatment and associated services (International Council of Nurses [ICN] 2021:9), and nurse anaesthetists’ critical role is recognised in both developed and developing countries; nurses have been providing anaesthesia services in the United States of America (USA) since the 1800s (Edgcombe et al. 2019:1).

South Africa is a developing country with a deficit of anaesthesia providers; the country currently has a physician anaesthesia provider ratio of 3.3 per 100 000 population (WFSA 2021:2). The government is thus in the process of developing and implementing a National Surgical Obstetric and Anaesthesia Plan (NSOAP) (Hardcastle & Chu 2020:176). The effects of inadequate access to surgical and anaesthesia services are significant and attributed to inequalities in healthcare system resources between the well-resourced private health sector and the overburdened, understaffed and less-equipped public health sector (Holtzhausen et al. 2021:672). Inequality in healthcare resources also exists between the under-resourced rural areas and better-resourced urban areas of South Africa (Maphumulo & Bhengu 2019:4). Moreover, the reality of the shortage of physician anaesthesia providers is potentially worse as data do not fully reflect healthcare access because of a disparity in the distribution of anaesthesia providers between public and private health sectors and the under-resourced rural and better-resourced urban areas (Tiwari, Chikte & Chu 2021:2). The shortage of anaesthesiologists in South Africa is also compounded by factors such as migration and universities training fewer anaesthesiologists to meet the increasing demand (Majiet 2018:n.p).

Furthermore, the health and well-being of the growing South African population are threatened by the current high burden of trauma, communicable and non-communicable diseases, and maternal and child health challenges, many of which require surgical and anaesthesia care (Van Straten et al. 2017:788). Holtzhausen et al. (2021:672) highlighted the impact of limited anaesthesia services in rural areas of South Africa, and they argued that increasing the accessibility and availability of caesarean sections (where clinically indicated) can help South Africa reduce its maternal mortality ratio from 134 deaths per 100 000 live births to 70 deaths per 100 000 live births.

The history of attempts to introduce nurse anaesthetists in South Africa dates back to the late 1960s and early 1970s (Nkuna 2016:9). Initially, there were discussions within the medical community about addressing the scarcity of anaesthesiologists by training non-medically certified individuals to administer anaesthetics under the supervision of a doctor. However, these proposals faced opposition, with concerns raised by South African Medical Association and the South African Medical and Dental Council and confusion surrounding the terminology used to describe the role of nursing personnel assisting anaesthetists. Eventually, a legislation was passed in 1974 to create a distinct category known as anaesthetic assistants, who underwent 2 years of training and could administer anaesthesia under the supervision of a doctor in designated hospitals (Nkuna 2016:9). Despite these efforts, concerns about medico-legal issues and the withdrawal of support from the Medical Defence Union hindered the establishment of nurse anaesthetists as an independent category. The issue was revisited in 1981 but was sent to an ad hoc sub-committee for further examination (Nkuna 2016:9).

Presently, anaesthesia is administered in South Africa only by medical practitioners (The South African Society of Anaesthesiologists [SASA] 2022:3). There is no formal recognition of the role of nurse practitioners in anaesthetics and the administration of anaesthesia, apart from being anaesthetic assistants. As assistants, they do not make any independent decisions regarding the provision of anaesthesia to the patient (Maharaj, Cronjé & Jithoo 2021:15). Moreover, a solution to the anaesthesia provider shortage partly requires involvement from the anaesthesiology community (Barash & Newton 2018:2). Their engagement is founded on the idea that evidence-based solutions should be implemented which are locally relevant and driven to expand access to surgical care (Hardcastle & Chu 2020:177).

### Problem statement

In South Africa, limited healthcare services impact patients’ welfare and their human rights, and increase the risk of preventable anaesthesia-related deaths. The country’s healthcare system challenges should compel authorities to seek and implement sustainable solutions to deliver quality and cost-effective healthcare services to underserved areas (Maphumulo & Bhengu 2019:7). Training advanced practice nurses to function as NPAs (as in other countries such as the USA) can improve South Africans’ access to anaesthesia services. There is also a need to have a generative conversation, especially with the anaesthesiology community (WFSA 2021:2), to critically engage them and seek their perspectives on the anaesthesia workforce shortage so that solutions are locally relevant. Based on the introduction and problem statement, the research question for this study was: ‘What are anaesthesiologists’ perspectives regarding the need for nurse anaesthetists in South Africa?’

### Definition of key concepts

#### Anaesthesiologist

An anaesthesiologist is a specialist doctor who provides anaesthesia services independently to all patients (The SASA 2022). In this study, an ‘anaesthesiologist’ referred to an anaesthesia specialist doctor from whom the researcher sought perspectives regarding the need for nurse anaesthetists in South Africa.

#### Need

A ‘need’ refers to a condition requiring supply or relief to reach a desirable condition (Merriam-Webster Dictionary 2021). In this study, ‘need’ referred to the necessity for nurse anaesthetists to act as anaesthetic providers as a possible solution to the shortage of anaesthesiologists in South Africa.

#### Nurse anaesthetist

A nurse anaesthetist refers to a licensed professional nurse who has undergone an extensive, accredited anaesthesia training programme and is certified by a relevant statutory body to administer anaesthesia services (American Association of Nurse Anaesthetists 2021:1). In this study, a ‘nurse anaesthetist’ referred to a licensed professional nurse who had undergone an extensive, accredited anaesthesia training programme, but they did not have a practice in South Africa.

#### Perspective

Perspective refers to the viewpoint of a participant in the subject domain (Clarke & Davison 2018:1). In this study, ‘perspective’ referred to the anaesthesiologists’ views regarding the need for nurse anaesthetists in South Africa.

## Research methods and design

This study used a qualitative and contextual research design and an exploratory and descriptive method.

### Setting

The study was conducted in South Africa, and it included participants from Gauteng, Western Cape, Mpumalanga and Eastern Cape provinces.

### Population and sampling

This study’s population comprised anaesthesiologists registered with the Health Professions Council of South Africa (HPCSA). The target population comprised anaesthesiologists who were willing and consented to participate. The accessible population was derived from a list of 1364 full members of the SASA, with at least 5 years of experience. A purposive sampling technique coupled with snowball sampling was used to select participants. The study’s sample size of 13 participants was determined by data saturation and confirmed by an independent coder.

### Inclusion and exclusion criteria

The following inclusion criteria were used during the sampling process:

- Participants were male or female anaesthesiologists practising at public and private hospitals in South Africa.
- They had to have 5 years’ experience in the field.
- They had to be willing and consent to participate in the study.

The exclusion criteria were:

- Individuals with less than 5 years’ clinical experience.
- Individuals primarily working in non-clinician roles.

### Data collection

The researcher conducted a pilot interview to test the interview questions and then collected data over 7 months through individual, semi-structured interviews. The interviews were conducted face-to-face or via video conferencing, depending on the participant’s preference. The researcher gained access to participants by contacting the SASA, who then invited potential participants who met the study’s inclusion criteria. The researcher distributed a research information letter and consent form to participants before the interview commenced, which lasted 45–60 min. During the interview, participants were asked one open-ended question: *How do you see the need for nurse anaesthetists in South Africa?* The interviews were recorded, and the researcher took field notes.

### Data analysis

The data were analysed using Colaizzi’s (1978) seven-step descriptive method. Step 1 comprised familiarisation with the data. The researcher read each transcript at least three times and listened to each recording five times until an understanding of the anaesthesiologists’ perspectives was achieved. Step 2 entailed identifying significant statements; the researcher thus extracted significant statements related to participants’ perspectives from each transcript. These statements were grouped by transcript number, participant letter and entry number. In Step 3, the researcher derived meaning from all significant statements identified in Step 2. The researcher coded primary meanings and grouped them into separate categories to reflect an exhaustive description.

During Step 4, the researcher reviewed the formulated meanings and grouped them by relevance into cluster themes. They were then coded with a descriptive label that best described the participants’ perspectives. In Step 5, the researcher integrated all emergent themes into an exhaustive description of the anaesthesiologists’ perspectives. Step 6 condensed the exhaustive description into a short, dense statement that captured just those aspects deemed essential to the phenomenon’s structure. In Step 7, the researcher formulated a fundamental statement to fully describe the anaesthesiologists’ perspectives.

### Trustworthiness

Trustworthiness was ensured by adopting Lincoln and Guba’s (1985) criteria for evaluating qualitative research; these include credibility, transferability, dependability and confirmability. Credibility was ensured through prolonged engagement, peer review by the supervisors, triangulation and member checking. To promote transferability, the researcher carefully documented the entire research process, methodology and findings to enable other researchers to apply the findings in their contexts. To ensure dependability, the researcher transparently described the steps taken from the start of the research process to reporting on the findings. The researcher also kept the interview transcripts, recordings and field notes for auditing purposes. Confirmability was ensured by providing an audit trail highlighting every step of the data analysis process to offer a rationale for all decisions that were made.

### Ethical considerations

Permission to conduct the research was requested from the University of Johannesburg’s Faculty of Health Sciences Research Ethics Committee (reference number: REC-1309-2021), the Higher Degrees Committee and the SASA as the official body that keeps a register of anaesthesiologists in South Africa. This study was underpinned by the ethical principles of respect for persons, beneficence and non-maleficence and justice (Grove & Gray 2019:134).

The researcher adhered to the ethical principles of informed consent and anonymity, and participants’ rights were prioritised. No foreseeable risks existed, and participants held the power to withdraw from the interviews at any moment. Participants indirectly benefitted from voicing their opinions on the need for nurse anaesthetists. To avoid any harm, participants could stop the interview or leave the research before it began. Participation was also voluntary and fair, with participants receiving fair treatment and controlling the interview’s schedule and location.

## Results

The results were categorised into a description of the study’s sample, themes and categories.

### Description of the study sample

The study’s sample comprised 13 participants, of which seven were male and six were female. All the participants were anaesthesiologists registered with the HPCSA as specialist anaesthesiologists. The participants had diverse experience and fields of anaesthesiology practice within South Africa. The years of experience among participants ranged from 6 to 44 years. The participants’ practice fields encompassed various domains, including private hospitals, academic institutions and public hospitals, geographically spread among urban areas in Gauteng, the Western Cape, and Mpumalanga and rural areas in the Eastern Cape.

### Themes and categories

The major themes and categories that emerged from the data are described in Table 1.

**TABLE 1:** Major themes and categories.

Themes	Categories
Theme 1: Participants’ perspectives on the need for nurse anaesthetists varied	1.1 Some participants admitted and recognised the great need for nurse anaesthetists. 1.2 The participants greatly supported the concept of one anaesthesiologist as a supervisor; some visualised a perfect scenario. 1.3 Some participants resisted the idea of an independent nurse anaesthetist and stated their deep concerns and need for nurse anaesthetists to act as their assistants.
Theme 2: The specialist fraternity should compile the nurse anaesthetists’ scope of practice, which is extremely important to accurately guide nurse anaesthetists and ensure regulation	2.1 The participants concurred on a very strict scope of practice for nurse anaesthetists to practise legally and be protected from litigation. 2.2 Supervision by anaesthesiologists was a priority to work independently, and conditions and criteria for nurse anaesthetists should be subjected to the conditions set in the scope of practice. 2.3 Regulatory bodies should manage nurse anaesthetists, and it was highly recommended that nurse anaesthetists be registered before they are allowed to practise.
Theme 3: Nurse anaesthetists’ training should be comprehensive and at the same level as diplomate anaesthetists’ training, and should be led by specialist anaesthesiologists with the support of nursing lecturers	3.1 A team effort under the leadership of anaesthesiologists would be essential to manage the curriculum, training, assessments and supervision. 3.2 The subjects and skills that needed to be included were basic sciences, medical sciences and general medical knowledge. 3.3 Nurse anaesthetists should demonstrate highly developed technical skills, profound comprehension of the process, and insight into potential risks that could develop in order to practise independently.

*Source*: Yezu, K.K., 2023, ‘Anaesthesiologists’ perspectives of the need for nurse anaesthetists in South Africa’, Master’s thesis, Department of Nursing, University of Johannesburg, Johannesburg, viewed n.d., from https://hdl.handle.net/10210/512174

#### Theme 1: Participants’ perspectives on the need for nurse anaesthetists varied

While participants acknowledged the potential benefit of nurse anaesthetists addressing the shortage of specialists, particularly in remote areas, their views were divided. Some argued that nurse anaesthetists with adequate training and supervision could effectively support anaesthesiologists and expand access to surgical care. Others expressed concerns about patient safety and the appropriateness of nurse anaesthetists practising independently. Additionally, potential challenges were highlighted, including opposition to the concept because of its novelty, scope of practice, medico-legal considerations, financial implications and inherent risks. These diverse perspectives suggest the need for careful consideration and a comprehensive approach when exploring the role of nurse anaesthetists in South Africa’s healthcare system. The following participant quotes support this theme:

‘In South Africa, mostly in the remote areas, in the rural areas the need is there to train people to do an anaesthesia because there is a need. Let me just put it like this.’ (Particpant B, African male, 36 years)

‘If it is introduced, I do think anaesthetists should take the role of a supervisor and nurse anaesthetists should work under anaesthetists or anaesthesiologists and not totally on their own. There should be someone on the floor, in a theatre complex. It is an anaesthesiologist on the floor in a theatre complex. And they should be able to call anaesthetists to assist if they land into trouble and it’s the same way diploma anaesthetists or diplomats as we call them.’ (Participant E, white female, 32 years)

‘It is not a person that I see that can independently do an anaesthetic from beginning to end.’ (Participant F, white female, 14 years)

#### Theme 2: The specialist fraternity should compile the nurse anaesthetists’ scope of practice, which is extremely important to accurately guide nurse anaesthetists and ensure regulation

Participants emphasised the need to develop nurse anaesthetists’ scope of practice and consider their job descriptions to ensure these individuals are legally fit to practise. The scope and regulation of practice were also underscored to ensure high standards and qualifications to promote safe and competent practice. Ultimately, nurse anaesthetists’ scope of practice has to be compiled collaboratively, with specialist anaesthesiologists taking the lead. The practice’s regulation by various bodies is also recommended to ensure accountability and adherence to professional standards. The following quotes support this theme:

‘First, they have to develop a guideline clearly stipulating and mentioning exactly what the scope guideline is, so that will have to be obviously done by a panel of specialists and especially those who are going to be training these people in the academic centres … so I think it will have to be a panel of people to come up with the scope of practice.’ (Participant D, African male, 6 years)

‘Anaesthesia is a complex skill and when you are doing very simple cases, the simplicity in terms of the patients themselves being healthy and the surgical procedure itself being simple and then one could argue that then you could allow a less skilled individual to conduct that anaesthetic. However, even in those simple cases, complications can arise. I think my concern around allowing nurses to work on their own without supervision is that should they find themselves in a situation like that. The ideal situation is that there should be on-site supervision, but obviously now, resource constrained environment this isn’t possible. So, I just feel like we can’t compromise patient care by offering a service where if there’s a complication … So yeah, by supervision I just mean someone on-site or someone on the telephone, ideally on-site.’ (Participant C, African female, 13 years)

‘I would think that they would be registered with both the SANC as well as HPCSA.’ (Participant E, white female, 32 years)

#### Theme 3: Nurse anaesthetists’ training should be comprehensive and at the same level as diplomate anaesthetists’ training, and should be led by specialist anaesthesiologists with the support of nursing lecturers

Participants emphasised the importance of a well-structured, formalised training curriculum for nurse anaesthetists. The training must be comprehensive and cover various aspects of anaesthesia, including theoretical knowledge, practical skills and exposure to the theatre complex. Specialist anaesthesiologists should be involved, particularly in simulating various surgical cases. The training was also recommended to be robust and equivalent to the postgraduate diploma in anaesthesiology for medical practitioners. The following quotes support this theme:

‘I strongly believe that anaesthesiologists should be involved in the training when it comes to simulation scenarios where we put the built-in app of the simulation scenario, or their opinion and their input of the anaesthesiologists of what cases should be elaborated, where the need of the anaesthetic nurse is vital. It should be set up and designed by anaesthesiologists.’ (Participant G, white female, 29 years)

‘But I would definitely think the basics that would be needed: some understanding of anatomy, some understanding of physiology and pharmacology, some basic knowledge of that, and then so like maybe the first year is basic anatomy, physiology, pharmacology and then there afterwards, the practicalities of surgical procedures. And taking those three basic sciences and applying them practically to do the job.’ (Participant M, African female, 14 years)

‘So, you need to present candidates that are reliable like that. And you need to, I think you need to follow the American system. You’ll need to present us with candidates that we are not we’re not fixing the mistakes all the time.’ (Participant L, white male, 28 years)

## Discussion

The study’s findings revealed there is a recognition of the need for nurse anaesthetists, particularly in remote and underserved areas of South Africa. This need is based on the scarcity of specialist anaesthesiologists. These perspectives align with the broader discourse on healthcare equity and access, where nurse anaesthetists can serve as valuable contributors increasing access to surgical and anaesthesia services (Wollner et al. 2020:924). Therefore, well-trained nurse anaesthetists’ role in addressing the existing healthcare disparities is also recognised. These healthcare disparities, as seen mainly in Africa’s rural areas, including South Africa and Southeast Asia, restrict essential procedures like caesareans, potentially hindering reductions in maternal mortality rates (Holtzhausen et al. 2021:672; Turkot & Banks 2019:1). Other countries like Kenya, Burundi, the Democratic Republic of Congo, Ethiopia, Ghana, Sierra Leone, Uganda and Somaliland have made good progress in increasing access to safe anaesthesia care by task-shifting and training NPAs to deliver certain forms of anaesthesia (Edgcombe et al. 2019:2; Rowles & Meeusen 2021:n.p). Barash and Newton (2018:1) also recognised nurse anaesthetists as one possible solution to the anaesthesia workforce shortage in Africa (Barash & Newton 2018:1).

High-income countries such as the USA, Sweden and France have successful anaesthesia delivery models that involve nurse anaesthetists collaborating with anaesthesiologists (Tenedios et al. 2018:161). In South Africa, there is a similar need to train nurse anaesthetists in order to complement overburdened medical practitioners, while contributing positively to the welfare of patients (DENOSA 2012:1). A recommendation is for these healthcare providers to work under a clearly defined scope of practice (DENOSA 2012:1).

Presently, in South Africa, anaesthesia is only administered by medical practitioners (The SASA 2022:3). There is no formal recognition of the role of nurse practitioners in anaesthetics and the administration of anaesthesia, apart from being anaesthetic assistants who work under the direct supervision of medical practitioners (Maharaj et al. 2021:15). Anaesthetic nurses in KwaZulu-Natal favoured supervision from anaesthetists over their nursing colleagues because they believed anaesthetists had more confidence in their knowledge and skills than their nursing peers (Maharaj et al. 2021:15). The concept of supervision in anaesthesia is also endorsed internationally, where it is recommended that whenever possible, anaesthesia should be led by an anaesthesiologist (Gelb et al. 2018:2047). Furthermore, if someone other than a physician anaesthesiologist is administering anaesthesia, they should be under the direction and supervision of an anaesthesiologist (Gelb et al. 2018:2047).

Supervising other anaesthesia providers is a distinctive skill, and when performed by an anaesthesiologist, it holds a pivotal role in delivering high-quality patient care and fostering anaesthesia trainees’ professional growth. The term ‘supervision’ encompasses all the clinical oversight functions aimed at ensuring the quality of clinical care whenever the anaesthesiologist is not the sole anaesthesia care provider (Perez et al. 2019:2). It is also highlighted that effective supervision in the context of clinical care ensures the quality and safety of both the patient and the trainee. Supervision involves a structured approach that includes the gradual delegation of responsibilities, ongoing evaluation and the exchange of clinical judgement (Perez et al. 2019:2).

Resistance to nurse anaesthetists in South Africa is historical; the idea of nurse anaesthetists was opposed because of medico-legal issues when the Medical Defence Union stated that it would withdraw support for actions involving an anaesthetic assistant (Nkuna 2016:10). Moreover, resistance to the idea of independent nurse anaesthetists is also not unique to South Africa. Similar concerns have been raised in countries such as India, where the medical community cited concerns about patient safety if anaesthesia was given by non-physician anaesthesia providers. Similarly, nurse anaesthetists have been excluded from anaesthesia practice in Canada for medico-legal reasons (Tenedios et al. 2018:161). The Canadian Anaesthesiologists’ Society (CAS) recently opposed the introduction of certified registered nurse anaesthetists (CRNAs) as independent anaesthesia providers as a solution for improving peri-operative access. It was argued that anaesthesia remains physician-led with the support of assistants, using a principle of delegation, not substitution (CAS 2022:1).

Similarly, in Australia, the majority of specialist anaesthetists in a single-centre university hospital opposed an anaesthesia-led nurse practitioner model for low-risk colonoscopy patients (Weinberg et al. 2020:1). They cited concerns about patient safety, deviations from the existing guidelines set forth by the Australian and New Zealand College of Anaesthetists regarding procedural sedation, and practical feasibility issues, hindering the adoption of this model (Weinberg et al. 2020:1). High standards of training in anaesthesia are important for three fundamental reasons: unsafe anaesthesia may lead to poor outcomes with increased morbidity and mortality, low-quality training paints a poor image of anaesthesia practice and those poorly trained are likely to operate beyond their scope of practice leading to poor patient outcomes (Vreede et al. 2019:1199).

As highlighted earlier, there is no formal recognition of the role of nurse practitioners in anaesthetics and the administration of anaesthesia in South Africa. A scope of practice is also currently non-existent (Maharaj et al. 2021:15). Nurse anaesthetists’ role and scope of practice have been primarily defined with strong reference to the International Federation of Nurse Anaesthetists (IFNA) and the ICN as authoritative bodies in the field of nurse anaesthesia practice (ICN 2021:14). According to the IFNA, nurse anaesthetists’ scope of practice entails the provision of anaesthesia management and care during the peri-operative period (Herion et al. 2019:407). This period involves the delivery of anaesthesia care before, during and after surgery. The nurse anaesthetist conducts patient assessment and monitoring, and prepares and inspects equipment and materials used during the peri-operative period (Nagelhout & Elisha 2018:15). The nurse anaesthetist also prepares and administers general and locoregional anaesthesia drugs to patients of all demographics and according to the type of surgical intervention (IFNA 2016:10). The scope of practice of NPAPs throughout Africa is overall similar (i.e. the complete scope of independent practice) (Law & Bulamba 2019:4).

Countries ultimately develop their own scope of practice for nurse anaesthetists, either based on the IFNA’s standards in collaboration with its member countries or by adopting standards related to anaesthesia activities. These competencies are designed to support and define the roles and responsibilities of nurse anaesthetists (ICN 2021:16).

The participants’ recommendation that nurse anaesthetists handle uncomplicated cases (American Society of Anaesthesiologists [ASA] level I or II) with oversight from an anaesthesiologist for complex cases, aligns with the current anaesthesia practice in South Africa. Diplomate anaesthetists (doctors with a diploma in anaesthesia and a minimum of 6 months of supervised practice in an accredited institution) are thus eligible to independently practise general and regional anaesthesia in ASA class I and II patients, and under supervision of a specialist anaesthesiologist for ASA class III patients (SASA 2022:4). This concept of a supervised and collaborative model of anaesthesia care is also present in Sweden, where the registered nurse anaesthetist (RNA) can independently induce, complete and carry out general anaesthesia according to specified protocols and under the supervision of an anaesthesiologist for ASA class I or II patients, and for ASA III cases or higher, the RNAs plan and administer general anaesthesia in collaboration with an anaesthesiologist (Rönnberg et al. 2022:56). Similarly, in the United Kingdom, physician assistants (anaesthesia) (PA[A]) provide general, regional and local anaesthesia under the supervision of a physician anaesthetist (Tenedios et al. 2018:161). In France, nurse anaesthetists work under a physician anaesthetist, administer general and regional anaesthesia (reinject local anaesthetic through a device placed by an anaesthesiologist) and participate in peri-operative resuscitation (Tenedios et al. 2018:161).

According to the IFNA, part of the scope of practice for nurse anaesthetists is to recognise and take appropriate action when complications occur. They must immediately consult with appropriate others if patient safety requires intervention or if the incidence exceeds their scope of practice (ICN 2021:18). In South Africa, the requirement for patients to consent for anaesthesia to be administered by nurse anaesthetists is consistent with international standards of ethics in healthcare. It is thus a mandatory practice for anaesthesia providers in South Africa to obtain informed consent before administering anaesthesia (SASA 2022:3). In South Africa, diplomate anaesthetists are required to disclose to the patient that they are not specialist anaesthesiologists (SASA 2022:3). By obtaining separate consent for anaesthesia administered by nurse anaesthetists, patients have an opportunity to make an informed decision about their care. The determination of what constitutes qualified anaesthesia providers and the type of cases for which they are equipped (i.e. scope of practice) is complicated and must be determined by the national or state anaesthetic society and the relevant Ministry of Health (Law et al. 2019:965).

The findings also revealed a consensus among anaesthesiologists that regulatory bodies should manage and control nurse anaesthetists’ practice. This perspective aligns with the broader international healthcare context, where regulatory oversight is considered essential to ensure patient safety and maintain the highest standards of care (Leslie et al. 2021:1). The IFNA’s educational standards also advise that certification, credentialling and regulation for nurse anaesthetists are important as it provides formal recognition, authorises a legal scope of practice, allows legal use of a title, and explicates standards of practice (ICN 2021:18). In support of this view, non-physician anaesthesia providers’ training must be recognised by the government and medical professional bodies (Kapoor 2019:963). It is also recommended that anaesthesia be guided by a set of locally agreed-upon practice standards (Vreede et al. 2019:1199). This ensures that healthcare practitioners, including nurse anaesthetists, are well-equipped to provide safe and effective care to patients (ICN 2021:18).

A strong recommendation is that nurse anaesthetists should be officially registered with either South African Nursing Council (SANC), HPCSA or the College of Anaesthetists of South Africa before they are allowed to practise. This perspective is grounded in the belief that registration serves as a fundamental step in ensuring accountability and adherence to professional standards. It also acts as a safeguard against unqualified practitioners entering the field (Vreede et al. 2019:1199). This study’s findings ultimately highlight anaesthesiologists’ perspectives regarding the need for regulatory oversight and registration of nurse anaesthetists. Their views add to the understanding that regulatory bodies play a pivotal role in ensuring accountability, adherence to professional standards and the safe practice of nurse anaesthetists. Additionally, the guidance provided by the regulatory bodies contributes to the ongoing improvement of healthcare quality and patient safety.

The findings also emphasised the significance of collaboration among experts in the field of anaesthesiology to develop a comprehensive curriculum for nurse anaesthetist training. This collaborative approach aligns with the concept of interprofessional education (IPE) and collaborative practice, which emphasises the importance of healthcare professionals working together to improve patient outcomes (Van Diggele et al. 2020:1). Interprofessional education has been found to promote interprofessional cooperation between the medical and the nursing profession (Homeyer et al. 2018:10). It is also found to promote teamwork among anaesthesia professionals (Khalafi, Sarmeydani & Akhoondzadeh 2023:105).

The training of nurse anaesthetist should be equivalent to the postgraduate diploma in anaesthesiology for medical practitioners. This finding is similar to the IFNA’s educational standards for preparing nurse anaesthetists, which can range from 24 to 36 months, encompassing didactic coursework, clinical experience and assessments (ICN 2021:18). Nurse anaesthesia education must be of sufficient length to allow for a rigorous didactic and clinical curriculum that prepares students to master anaesthesia competencies (ICN 2021:18). Successful training of non-physician anaesthesia providers ultimately requires an adequate volume and variety of surgeries, appropriate equipment and a structured and comprehensive programme to develop the adaptability and problem-solving skills necessary to handle a variety of clinical scenarios effectively (Kapoor 2019:963).

Anaesthesiologists’ involvement in educating nurse anaesthetists also offers some advantages. Their expertise in critical analysis, ability to enhance academic rigour and role in fostering trust and understanding between the two professions are all recognised as essential for advancing the field of nurse anaesthesia (Swerdlow, Osborne-Smith & Berry 2020:997). Similarly, anaesthesiologists need to take a lead role in developing the non-physician anaesthesia provider workforce by proposing, designing, guiding and conducting appropriate training programmes to ensure that quality and safety standards are met (Kapoor 2019:963). Anaesthesiologists’ supervision and clinical instruction of nurse anaesthetists in operating rooms also allow nurse anaesthetists to practise at the specified quality standard (Swerdlow et al. 2020:997). If newly qualified anaesthesia providers meet situations for which they have not been trained, problems quickly ensue (Edgcombe et al. 2019:1).

The study’s findings highlighted the fact that collaborative effort is required under the leadership of anaesthesiologists to establish a robust training programme for nurse anaesthetists. The participants’ perspectives aligned with international standards and best practices in nurse anaesthetist education. By drawing upon existing literature from different countries, this discussion underscores the importance of nurse anaesthetists as valuable members of the healthcare team and advocates for their comprehensive training to ensure patient safety and quality care.

The inclusion of subjects like anatomy, physiology, pharmacology, medical sciences and general medical knowledge also aligns with international best practices in nurse anaesthetist education. The standard curriculum for nurse anaesthetists developed by the IFNA is an educational programme that prepares nurse anaesthetists to provide safe anaesthesia care in the peri-operative period. Key components include anatomy, physiology, pathophysiology, pharmacology, chemistry and physics in anaesthesia, general and regional anaesthesia techniques, monitoring and medical devices, patient assessment and management, anaesthesia techniques for different patient populations, resuscitation and life-saving procedures, fluid and blood therapy and pain management. This curriculum ensures that nurse anaesthetists are well-trained to deliver high-quality care and contributes to patient safety during surgical procedures (ICN 2021:40). It has also been previously emphasised that anaesthesia is inherently complex and potentially hazardous, and its safe provision requires a high level of expertise (Gelb et al. 2018:2047).

The participants similarly emphasised the significance of nurse anaesthetists possessing advanced technical skills. This aligns with the global consensus of the WHO’s International Standards for Safe Practice of Anaesthesia, stating that anaesthesia requires highly skilled practitioners (Gelb et al. 2018:2047). The IFNA also recognises that nurse anaesthetists are trained to provide services focusing on safety and patient outcomes, which necessitates a high level of technical proficiency. Similarly, the ICN states that nurse anaesthesia requires complex decision-making skills and clinical competencies. Non-technical skills, such as situational awareness, decision-making and teamwork contribute to safer procedures by avoiding errors (Radhakrishnan et al. 2022:64).

Continuous skill development among nurse anaesthetists is an essential component of maintaining and improving technical competence (Jeon et al. 2020:1). Simulation in nurse anaesthesia continuing education can also improve clinical skill and adequately prepare providers for emergency resuscitation (Gabbard & Smith-Steinert 2021:65). Nurse anaesthetists must always have a plan, be prepared to make quick decisions in cases of emergency and be one step ahead of challenges (Rönnberg et al. 2022:56). This means that anaesthetists require insight into potential risks and understand how to handle them effectively.

These findings emphasise the vital role of nurse anaesthetists in areas with a shortage of specialist anaesthesiologists, highlighting the need for clear roles, supervision and a well-defined scope of practice. Comprehensive training programmes aligned with international standards are required, focusing on essential educational components like basic and medical sciences, emergency response and technical proficiency. Collaboration with anaesthesiologists is ultimately crucial to ensure quality care and patient safety, outlining the necessity for continuous education and adherence to global expectations and qualifications for nurse anaesthetists.

### Limitations of the study

The study’s findings may not apply to other countries or healthcare systems because the study focused solely on anaesthesiologists in South Africa. Participants were also required to have at least 5 years of experience as specialist anaesthesiologists, potentially biasing the results towards more experienced individuals. Additionally, the non-existence of the nurse anaesthetist profession in South Africa means the recommendations based on this study may require further exploration.

### Implications of the study

Implementing nurse anaesthetists in South Africa has the potential to improve access to anaesthesia services in remote and underserved areas, addressing critical healthcare disparities. The study underscores the need for formal recognition, certification and regulatory frameworks for nurse anaesthetists to ensure safe and standardised practices. It highlights the importance of developing comprehensive training programmes for nurse anaesthetists, including collaboration with anaesthesiologists, to ensure competency and patient safety.

### Recommendations

The following recommendations are offered for nursing practice and policy development, education and further research.

#### Recommendations for nursing practice and policy development

- A joint committee of anaesthesia experts and the Department of Health should draft a regulatory framework for the nurse anaesthetist profession in South Africa to define educational requirements, scope of practice and professional standards necessary for certification and licensure of nurse anaesthetists.
- A collaborative approach is required involving various authorities such as SANC, HPCSA, SASA, the College of Anaesthesiologists of South Africa and the Department of Health to develop clear protocols for the supervision of nurse anaesthetists, especially in the initial stages of their practice if this programme is to be introduced in South Africa.
- There is a need to foster collaboration between nurse anaesthetists and anaesthesiologists. Mentorship programmes should be established, where experienced anaesthesiologists guide nurse anaesthetists during their practice.
- Resources should be allocated to develop and enhance healthcare infrastructure, particularly in rural and remote areas.
- Increased public awareness campaigns are required to educate society about nurse anaesthetists’ roles and capabilities.
- Engagement in dialogue should be promoted with the medical community, including anaesthesiologists, to address concerns and foster a better understanding of the potential benefits of nurse anaesthetists.

#### Recommendations for nursing education

- A pilot training programme should be initiated for nurse anaesthetists equivalent to the current postgraduate diploma in anaesthesiology for medical doctors in collaboration with healthcare institutions (SANC, HPCSA, SASA and the College of Anaesthesiologists of South Africa) and universities.
- A preliminary curriculum framework should be developed based on international standards. It should be adapted to include fundamental subjects like basic sciences, medical knowledge and practical skills, aligning with specific healthcare requirements in South Africa.
- Investments are required in building existing nursing educators’ capacity through training-of-trainers programmes to ensure a pool of qualified faculty members capable of delivering high-quality education to future nurse anaesthetists.
- Full training and education programmes should be introduced for nurse anaesthetists.

#### Recommendations for nursing research

Resources should be allocated for research initiatives to evaluate the effectiveness of nurse anaesthetist pilot programmes.

Comprehensive feasibility studies should be conducted to assess the practicality and potential challenges of introducing the nurse anaesthetist profession in South Africa.

## Conclusion

This study highlighted varied perspectives of anaesthesiologists on the need for nurse anaesthetists in South Africa. Views varied from welcoming independent, well-trained nurse anaesthetists practising under supervision or as assistants to the anaesthesiologists, to views completely opposing and resisting nurse anaesthetists administering anaesthesia. It was proposed that the specialist fraternity compile the nurse anaesthetists’ scope of practice, which is extremely important to guide nurse anaesthetists, regulated by relevant regulatory bodies. It was suggested that nurse anaesthetists’ training be at the same level as diplomate anaesthetists’ training and led by specialist anaesthesiologists with the support of nursing lecturers. The research findings advocate for the systematic introduction and integration of nurse anaesthetists into South Africa’s healthcare system. Key recommendations include initiating pilot training programmes, developing a tailored curriculum aligned with international standards and building the capacity of nursing educators. Regulatory frameworks, collaboration with anaesthesiologists, clear supervision protocols and infrastructure development in remote areas are crucial aspects to consider. Public awareness campaigns and open dialogue within the medical community are emphasised, along with dedicated resources for continuous research and feasibility studies.
